# A Treatise on Practical Chemistry and Qualitative Inorganic Analysis

**Published:** 1885-09

**Authors:** 


A Treatise on Practical Chemistry and Qualitative Inorganic Analysis, adapted for use in the laboratories of colleges and schools, by FRANK CLOWES, D. Sc., Professor of Chemistry at the University College, Nottingham, with illustrations. From the Fourth English edition. Philadelphia: Lea Brothers & Co. 1885.

It seems only the other day that we received the previous edition of this work, and we are glad to have evidence that the hearty commendations which we then gave it, have been so seconded by the teachers of chemistry, that in so short a time a fourth edition of the book is called for. The present edition has been wisely revised, and some useful illustrations added. For the medical student, it furnishes a systematic, intelligible and convenient laboratory guide.

